# Supplementary material for: Content-rich biological network constructed by mining PubMed abstracts
Source: BMC Bioinformatics. 2004 Oct 8;5:147. doi: 10.1186/1471-2105-5-147 (PMC528731; doi:10.1186/1471-2105-5-147)
Supplement: Additional File 2 — The original results of the above study (non-essential files are deleted to keep the file size under the limit set by BMC bioinformatics). [file 1471-2105-5-147-S2.bz2 › chilibotAdditionalFile2/dip10/150ID8479754E545/html/CKS1_CCND1.html]

 


 **CKS1** and **CCND1** 
  
Found 8 abstracts in PubMed, retrieved 8.  
 

 What does Google say? 
 PDF only 
| .edu only 

---

**Interactive relationship** (e.g. stimulation, inhibition, etc)

**Stimulatory relationship**- ... overexpression of cdc28 protein kinase 1  [ **CKS1** ]   **CKS1**  blocks the inhibition of the cyclin D1  [ **CCND1** ]  CDK4 complex by the CDK inhibitor p27 Kip1.  Ref: 12605034 Diagn Mol Pathol, 2003
**Neutral relationship**

**Non-interactive relationship** (e.g. studied together, co-existance, homology, etc.)

- We used a battery of antibodies directed against the following cytokeratins CKs  [ **CKS1** ]  7, and 19, actin, cell adhesion proteins desmoplakins, desmogleins, markers of terminal epidermal differentiation filaggrin, involucrin and loricrin, markers of proliferation PCNA, MIB, K 6, a marker of endocytosis clathrin and markers of cell growth, transforming growth factor TGF alpha and B cell leukemia lymphoma 2 bcl  [ **CCND1** ]  2.  Ref: 8850247 Photodermatol Photoimmunol Photomed, 1995
